# United Kingdom: Political Developments and Data in 2021: Lockdown and Lobbying

**DOI:** 10.1111/2047-8852.12362

**Published:** 2022-06-24

**Authors:** ALIA MIDDLETON

**Affiliations:** ^1^ University of Surrey Guildford UK

## Abstract

Covid‐19 still dominated politics in the UK in 2021, with another national lockdown announced in England just days into the year. Schools and non‐essential shops closed for several months, and restrictions were not finally lifted until July after the roll‐out of the vaccine programme. The final few weeks of 2021 were populated with multiple reports of Covid restrictions being broken at Downing Street during the earlier lockdowns. This, along with the government seeking to protect an MP accused of breaking lobbying rules, saw a substantial decrease in the Prime Minister's popularity over the year. In October, Sir David Amess was murdered during a constituency surgery – the first such death since 2016.

## Introduction

Covid‐19 was still dominating politics in the UK in 2021, with another national lockdown at the start of the year; schools were closed and did not reopen for two months. By July, most restrictions were lifted; the same month saw the government achieve its target of offering all adults in the UK a first dose of a Covid vaccine. A substantial Cabinet reshuffle took place in September 2021, with some senior ministers departing, others moving to alternative positions and an influx of junior ministers. Matt Hancock, Secretary of State for Health and Social Care since 2019, left the government in June, with former Chancellor Sajid Javid returning to the Cabinet to replace him.

The year saw elections to the Scottish Parliament, the Welsh *Senedd* and the London Assembly. After the postponement of many local elections and the London mayoral contest the previous year, these were finally held in 2021. A new pro‐Scottish independence party was formed, which saw defections at Westminster, and there were substantial changes in leadership in the Democratic Unionist Party (DUP) and the Green Party.

The government had to deal with considerable difficulties towards the end of the year. First, lobbying by North Shropshire MP Owen Paterson triggered an investigation, which the government attempted to delay; and second, regarding allegations of breaches of Covid regulations in Number 10 itself during the 2020 lockdowns, known as ‘Partygate’. The year also saw the largest simultaneous quashing of convictions by the Court of Appeal in relation to miscarriages of justice. A total of 39 former postmasters who had been prosecuted by Royal Mail after faulty software incorrectly showed financial shortfalls were absolved in April 2021. Lastly, the year saw the UK's withdrawal from Afghanistan after 20 years, with the evacuation of 15,000 people.

## Election report

There were no national elections in the UK in 2021.

### Subnational elections

The Scottish Parliament election was held on 6 May, with the Scottish National Party (SNP) once again gaining the largest number of seats; they have now been the largest party in Scotland since 2007. However, they gained only one additional seat, meaning they were a single seat short of an overall majority. The party initially governed as a single‐party minority, but in August 2021 the party entered a power‐sharing agreement with the Scottish Greens, who had had their best ever performance, gaining eight MSPs. Both the Green Party's co‐leaders subsequently took up ministerial posts in the administration. Scottish Labour slumped to their worst defeat in the history of the devolved administration, although Anas Sarwar had only taken over the leadership shortly before the campaign began.

The Welsh *Senedd* election was held on the same day, and demonstrating the regional distinctiveness in territorial politics in Britain, it saw the continued dominance of Welsh Labour. The party gained one seat and continued in a single‐party majority government. The collapse of the UK Independence Party (UKIP) in the *Senedd* elections saw them lose all seven of their seats, with the Conservatives being the key beneficiary and gaining five seats overall. Only four parties are now represented in the *Senedd*: Labour, Conservatives Plaid Cymru and the Liberal Democrats. A variety of local contests were held across England, with the Conservatives gaining 235 councillors, while Labour retained the mayoralties of London and Greater Manchester, amongst others.

## Cabinet report

The current government took office on 13 December 2019 and is a single‐party majority government. There were substantial changes in the composition of the Cabinet in 2021. Data on the cabinet composition of Johnson II can be found in Table 1.

**Table 1:** Cabinet composition of Johnson II in the UK in 2021

Duration of Cabinet	Inception	12 December 2019	Dissolution	Still in office at the end of the year					
Period covered by table	From	1 January 2021	Until	31 December 2021					
Type of Cabinet	Single‐Party Majority								
A.	Party/gender composition on 1 January 2021			Seats in Cabinet		Seats held by women		Seats in Parliament	
				*N*	%	*N*	% of party	*N*	%
	Conservative			21	100.0%	4	19.0%	365	56.2%
	Totals			21	100.0%	4	19.0%	365	56.2%

B.	Composition of Johnson II Cabinet on 1 January 2021	
	See previous editions of the *Political Data Yearbook* for the UK or http://politicaldatayearbook.com	

C.	Changes in composition of Johnson II Cabinet during 2021		Outgoing minister	Outgoing date	Incoming minister	Comments
	Ministerial title					
	Were there any Cabinet changes?	Yes				
	Secretary of State for Foreign and Commonwealth Affairs		Dominic Raab (1974 male)	15 September 2021	Liz Truss (1975 female(	Cabinet reshuffle
	Deputy Prime Minister of the UK		–	15 September 2021	Dominic Raab (1974 male)	Cabinet reshuffle
	Secretary of State for Justice		Robert Buckland (1968 male)	15 September 2021	Dominic Raab (1974 male)	Cabinet reshuffle
	Lord Chancellor		Robert Buckland (1968 male)	15 September 2021	Dominic Raab (1974 male)	Cabinet reshuffle
	Chancellor of the Duchy of Lancaster		Michal Gove (1967 male)	15 September 2021	Steve Barclay (1972 male)	Cabinet reshuffle
	Minister of State for the Cabinet Office		Michael Gove (1967 male)	15 September 2021	Steve Barclay (1972 male)	Cabinet reshuffle
	Secretary of State for Health and Social Care		Matt Hancock (1978 male)	26 June 2021	Sajid Javid (1969 male)	Resignation
	Secretary of State for Business, Energy and Industrial Strategy		Alok Sharma (1967 male)	8 January 2021	Kwasi Kwarteng (1975 male)	Moved to other position
	President of COP26			8 January 2021	Alok Sharma (1967 male)	New position
	Secretary of State for Education		Gavin Williamson (1976 male)	15 September 2021	Nadhim Zahawi (1967 male)	Cabinet reshuffle
	Secretary of State for International Trade		Liz Truss (1975 female)	15 September 2021	Anne‐Marie Trevelyan (1969 female)	Cabinet reshuffle
	Secretary of State for Housing, Communities and Local Government		Robert Jendrick (1982 male)	15 September 2021	–	Department renamed
	Secretary of State for Levelling Up, Housing and Communities		–	15 September 2021	Michael Gove (1967 male)	Department renamed
	Secretary of State for Digital, Culture, Media and Sport		Oliver Dowden (1978 male)	15 September 2021	Nadine Dorries (1957 female)	Cabinet reshuffle
	Minister of State for the Cabinet Office		Michael Gove (1967 male)	21 April 2021	Steve Barclay (1972 male)	Cabinet reshuffle

D.	Party/gender composition on 17 December 2021			Seats in Cabinet		Seats held by women		Seats in Parliament	
				*N*	%	*N*	% of party	*N*	%
	Conservative			21	100.0%	5	24.0%	361	55.5%
	Totals			21	100.0%	5	24.0%	361	55.5%

Note: All ministerial changes affected members of the Conservative Party.

*Source*: UK Parliament (n.d.).

On 8 January the Secretary of State for Business, Energy and Industrial Strategy, Alok Sharma, left his post to concentrate on his appointment as President of COP26, the United Nations Climate Change conference taking place later in the year in Glasgow. He still attended Cabinet in his new role. He was replaced in his original post by Kwasi Kwarteng, a junior minister in the department. Matt Hancock resigned as Secretary of State for Health and Social Care on 26 June (Hancock 2021), after CCTV footage was released by *The Sun* newspaper of him embracing an aide with whom he was in an extramarital relationship. The date of the video demonstrated that it was filmed prior to the lifting of Covid regulations enabling members of more than one household to mix, and as such Hancock's actions had been in breach. He was replaced by former Chancellor Sajid Javid.

A Cabinet reshuffle took place on 15 September, with a substantial number of junior ministers moved and many new junior ministers introduced to ministries for the first time. At the top level, the reshuffle saw some notable departures from Cabinet as well as demotions. Gavin Williamson, Secretary of State for Education, was removed from his post after two years of controversy over examination grades. Robert Jendrick was removed as Housing, Communities and Local Government Minister. Robert Buckland, who served as Justice Secretary and Lord High Chancellor of Great Britain, was dismissed from Cabinet after three years. Dominic Raab was moved from Foreign Secretary to take over both posts from Buckland, but also to occupy the revived post of Deputy Prime Minister, which had not been used since the end of the David Cameron–Nick Clegg Conservative–Liberal Democrat coalition in 2015. His removal as Foreign Secretary came amid criticism of his slow response to the Afghanistan crisis (Vaughan 2021). Liz Truss moved from the Department for International Trade to become the first Conservative female Foreign Secretary. Michael Gove moved to head the renamed Department for Levelling Up, Housing and Communities, including the ministerial brief for intergovernmental relations.

## Parliament report

Sir David Amess, MP since 1997 for Southend West (he had previously represented Basildon since 1983), was murdered on 15 October while holding a constituency surgery. It was the first time in over five years that an MP had been murdered in the UK. His death left the seat vacant and, due to the extraordinary circumstances of his death, the resulting by‐election was postponed to 2022. Amess had campaigned for many years for Southend‐on‐Sea to be awarded city status, which was bestowed by the queen as a memorial to him shortly after his death (Southend‐on‐Sea Council 2021). Data on the party and gender composition of the House of Commons can be found in Table 2.

**Table 2:** Party and gender composition of the House of Commons in the UK in 2021

			1 January 2021				31 December 2021			
			All		Women		All		Women	
Party			*N*	%	*N*	%	*N*	%	*N*	%
Conservative			365	56.2%	87	23.8%	361	55.5%	87	24.1%
Labour			200	30.8%	103	51.5%	199	30.6%	103	51.8%
Scottish National Party		(SNP)	47	7.2%	15	31.9%	45	6.9%	1	2.2%
Liberal Democrats			11	1.7%	7	63.6%	13	2.0%	9	69.2%
Democratic Unionist Party		(DUP)	8	1.2%	1	12.5%	8	1.2%	1	12.5%
Sinn Féin			7	1.1%	2	28.6%	7	1.1%	2	28.6%
Independent			4	0.6%	2	50.0%	6	0.9%	2	33.3%
Plaid Cymru			3	0.5%	1	33.3%	3	0.5%	1	33.3%
Social Democratic & Labour Party			2	0.3%	1	50.0%	2	0.3%	1	50.0%
Alliance			1	0.2%	0	0.0%	1	0.2%	0	0.0%
Green Party			1	0.2%	1	100.0%	1	0.2%	1	100.0%
Speaker			1	0.2%	0	0.0%	1	0.2%	0	0.0%
Alba Party			0	0.0%	0	0.0%	2	0.3%	0	0.0%
Vacant			0	0.0%	0	0.0%	1	0.2%	0	0.0%
Totals			650	100.0%	220	33.8%	650	100.0%	208	32.0%

*Source*: UK Parliament (2021a).

At the start of 2021, four MPs were sitting as independents after having their party whips withdrawn. During the year, two more Conservative MPs had the whip withdrawn and joined them as independents: Imran Ahmad Khan, MP for Wakefield, who was suspended by the party in June after being charged for historic sexual assault; and Rob Roberts, MP for Delyn, who had the whip withdrawn in July amid allegations of sexual misconduct.

In total, six by‐elections were held in 2021. The first, in Hartlepool, was held on 6 May after Labour MP Mike Hill resigned over sexual harassment allegations. The seat had been held by Labour since its inception in 1974, but was gained by the Conservatives in a record‐breaking swing from Labour. Labour had been criticized for fielding a former MP from a neighbouring seat who had lost his seat in 2019 as their candidate. Two further by‐elections were held due to the incumbent MPs resigning to take up posts in the Scottish Parliament (Neil Gray for the SNP in Airdrie and Shotts) and as Mayor of West Yorkshire (Tracy Brabin for Labour in Batley and Spen), respectively; in both cases, the incumbent parties held the seats. The Chesham and Amersham by‐election was held on 17 June after the death of Cheryl Gillan, the Conservative MP for the seat since 1992 and a former Cabinet minister, serving as Secretary of State for Wales under David Cameron, and Chair of the 1922 Committee during the 2019 Conservative leadership election. The Buckinghamshire seat had been held by the Conservatives since February 1974, but was gained by the Liberal Democrats. The death of another former Cabinet Minister, James Brokenshire, in October triggered another by‐election in Old Bexley and Sidcup on 2 December. In this case, the Conservatives held the seat, albeit with a reduced majority. The final by‐election of the year occurred in Shropshire North amid much controversy. The MP since 1997, Owen Paterson, a former Cabinet minister under David Cameron, was found to have broken lobbying rules by the Parliamentary Commissioner for Standards. A 30‐day suspension was recommended, which would have automatically triggered a by‐election contest. However, a whipped Conservative amendment to the parliamentary motion that sought to review the standards process was tabled (BBC 2021a), pausing the case. This proved to be controversial, and after protests by Conservative MPs, a boycott by Labour and the SNP, and plunging poll ratings, Johnson withdrew his support for Paterson, who resigned. The seat, held by the Conservatives since its inception in 1983, was lost to the Liberal Democrats.

The SNP saw the defection of two MPs, Kenny MacAskill and Neale Hanvey, to the newly formed Alba Party, headed by former Scottish First Minister Alex Salmond on 26 March 2021.

### House of Lords

New entries to the Lords included several former MPs, including Richard Benyon, who had had the Conservative whip withdrawn by Johnson in 2019 after voting against the government, and former junior ministers in the Brown administration, Gillian Merron and Vernon Coaker. Former Scottish Conservative leader, Ruth Davidson, became Baroness Davidson of Lundin Links. In an honours year heavy with former MPs and MEPs, Simon Stevens, Chief Executive of the National Health Service (NHS) until his elevation in July 2021, and Professor Sue Black, a forensic anthropologist were also awarded peerages. John Sentamu, former Archbishop of Canterbury and former Lord Spiritual, became a life peer. Data on the party and gender composition of the House of Lords can be found in Table 3.

**Table 3:** Party and gender composition of the House of Lords in the UK in 2021

			1 January 2021				31 December 2021			
			All		Women		All		Women	
Party			*N*	%	*N*	%	*N*	%	*N*	%
Conservative			258	32.5%	66	25.6%	258	33.1%	69	26.7%
Crossbench			180	22.7%	46	25.6%	191	24.5%	49	25.7%
Labour			177	22.3%	59	33.3%	168	21.5%	59	35.1%
Liberal Democrats			88	11.1%	32	36.4%	84	10.8%	32	38.1%
Non‐affiliated			49	6.2%	10	20.4%	39	5.0%	5	12.8%
Bishops			26	3.3%	5	19.2%	26	3.3%	5	19.2%
Democratic Unionist Party		(DUP)	5	0.6%	0	0.0%	5	0.6%	0	0.0%
Green Party			2	0.3%	2	100.0%	2	0.3%	2	100.0%
Ulster Unionist Party			2	0.3%	0	0.0%	2	0.3%	0	0.0%
Conservative Independent			1	0.1%	0	0.0%	1	0.1%	0	0,0%
Independent Labour			1	0.1%	0	0.0%	0	0.0%	0	0.0%
Independent Social Labour			1	0.1%	0	0.0%	1	0.1%	0	0.0%
Labour Independent			1	0.1%	1	100.0%	1	0.1%	1	100.0%
Lord Speaker			1	0.1%	0	0.0%	1	0.1%	0	0.0%
Plaid Cymru			1	0.1%	0	0.0%	1	0.1%	0	0.0%
Totals			793	100.0%	221	27.9%	780	100.0%	222	28.5%

*Source*: UK Parliament (2021b).

The Lords also saw the entry of Lord Cruddas amid considerable controversy. His appointment was rejected by the Lords Appointments Commission over a cash‐for‐access scandal during his time serving as Co‐Chair of the Conservatives (Scott 2022). However, the Prime Minister ignored the advice of the commission and approved his peerage; this was the first time a Prime Minister had acted despite the commission's recommendations since it was founded in 2000.

## Political party report

The pro‐Scottish independence Alba Party was launched in February, with former Scottish First Minister Alex Salmond taking over the leadership on 26 March. SNP MPs Neale Harvey and Kenny MacAskill defected to join the newly formed party on the same day, giving the Alba Party two seats in the House of Commons. Data on the changes in political parties can be found in Table 4.

**Table 4:** Changes in political parties in the UK in 2021

A.	Party leadership changes in 2021									
Alba Party	Became an officially registered party	Under the leadership of	Laurie	Flynn	1946	Male				
B.	Party leadership changes in 2021									
Alba Party	Laurie	Flynn	1946	Male	Resigned	26 March 2021	Alex	Salmond	1954	Male
Democratic Unionist Party (DUP)	Arlene	Foster	1970	Female	Resigned	28 May 2021	Edwin	Poots	1965	Male
DUP	Edwin	Poots	1965	Male	Resigned	30 June 2021	Jeffrey	Donaldson	1962	Male
Green Party	Jonathan	Bartley	1971	Male	Resigned	30 July 2021	Adrian	Ramsay	1981	Male
Green Party	Sian	Berry	1974	Female	Resigned	1 October 2021	Carla	Denyer	1985	Female

Note: The two Green Party leaders are co‐leaders.

*Sources*: Andrews (2021); Brooks (2021); and Green Party (2021).

The DUP saw considerable turbulence in their leadership. Their leader since 2015, Arlene Foster, resigned on 28 April after over 20 letters of no confidence were submitted over frustrations with her leadership. Her replacement Edwin Poots was elected amid rumours of bullying during his campaign. After nominating Paul Givan as First Minister despite opposition from the party, Poots was ousted after just 21 days in charge. Jeffrey Donaldson, MP for Lagan Valley since 1997 and a member of the Orange Order, was elected to replace him.

Both of the Green Party's co‐leaders, Jonathan Bartley and Siân Berry, stepped down in July, with Carla Denyer and Adrian Ramsey elected to replace them. After Richard Leonard's resignation as Scottish Labour leader on 14 January the party elected Anas Sarwar as his successor on 27 February. Sarwar is the first person of Asian descent and the first Muslim to lead a political party in the UK.

Polling for the Conservatives remained steady but low throughout much of 2021. While the Conservatives enjoyed a poll lead over Labour throughout much of the pandemic, by November 2021 the parties were level. Just a month later, amid the Paterson lobbying scandal and accusations of Covid regulations being breached in Downing Street, Labour moved into a 6‐point lead (YouGov 2021a). A separate poll (Survation 2021) showed that a general election held at this moment in time would have given Labour a majority, with half of Johnson's Cabinet losing their seats.

Much of this was also reflected in polling on the main party leaders themselves. Boris Johnson began the year with 55 per cent believing he was doing a bad job as Prime Minister. Despite a brief respite between March and June as the vaccine rollout commenced, Johnson closed the year with 71 per cent believing he was doing a bad job (YouGov 2021b). The Labour leader Keir Starmer did not directly reap the benefits of this, opening the year with 39 per cent saying he was doing a good job, but closing the year with polls showing 51 per cent of respondents thought he was doing a bad job (YouGov 2021c).

## Institutional change report

There were no major institutional changes made in the UK in 2021.

## Issues in national politics

After a Christmas dominated by increasing restrictions due to the new Delta variant of Covid‐19, the 4 January saw Johnson address the nation telling England to prepare for further restrictions to be announced, although children should return to school as planned. On 6 January, just a day after many schools returned after the Christmas break, a new national lockdown was announced. Schools remained closed until the start of March, and non‐essential shops began to reopen from April. By the end of March, the order to stay at home had been shifted to stay local instead. As part of his road map out of Covid, Johnson promised regular reviews on the lifting of restrictions. As such the planned lifting of most social restrictions at the start of June was delayed to 19 July. This allowed more time for the roll out of the vaccine programme. The end of November saw newspapers reporting on multiple instances of Downing Street staff breaking Covid restrictions on indoor gatherings the previous Christmas. Johnson told the Commons during Prime Minister's Questions that all guidance had been followed. However, December saw the leaking of a press conference rehearsal, during which the Prime Minister's Press Secretary, Allegra Stratton, a former journalist introduced to Number 10 in 2020, laughed and joked about a Christmas Party held in December 2020. Multiple breaches by staff were reported at 10 Downing Street, including by Johnson himself, accompanied by a photograph showing the alleged party, which Johnson described as a work event.

Royal Mail, the national postal service, prosecuted 736 branch managers of post offices between 2000 and 2014 for financial shortfalls in their branches. Horizon software, introduced by Royal Mail in 1999, contained glitches which provided inaccurate information on a branch's finances. Many postmasters, when the software presented them with shortfalls, used their own money to balance the books. Some went to prison; others were financially ruined and shunned by their communities. Many experienced family break‐ups, stress, addiction and one postmaster took his own life. Although settlements had been agreed with over 500 former postmasters in 2019 after a High Court ruling, April 2021 saw the convictions of 39 postmasters quashed on the same day after a review by the Court of Appeal (Peachey 2021). The judge described it as ‘an affront to the public conscience’. Paula Vennells, chief executive of Royal Mail from 2012 to 2019, stepped down from her roles as non‐executive director and as an Anglican priest the same day.

The Taliban retook Afghanistan in 2021 and gave 31 August as a deadline for American and British troops to withdraw, leading to the withdrawal of a British military presence for the first time in 20 years. Over 15,000 military personnel and civilians were evacuated by the UK ahead of the deadline (BBC 2021b). Those evacuated includes former Afghan UK staff and their families, who were potentially at risk from the new regime.

## References

[epdy12362-bib-0001] Andrews, C. (2021). DUP leadership: Sir Jeffrey Donaldson is only candidate. BBC News; online 22 June. Available online at: https://www.bbc.co.uk/news/uk‐northern‐ireland‐57560163

[epdy12362-bib-0002] BBC (2021a). Owen Paterson: Anger as Tory MP Avoids Suspension in Rule Shake‐Up. BBC News; online 3 November. Available online at: https://www.bbc.co.uk/news/uk‐politics‐59154221

[epdy12362-bib-0003] BBC (2021b). Afghanistan: How many People has the UK Evacuated? BBC News; online 7 December. Available online at: https://www.bbc.co.uk/news/uk‐58245684

[epdy12362-bib-0004] Brooks, L. (2021). Alex Salmond Launches New Independence‐Focused Alba Party. The Guardian 26 March. Available online at: https://www.theguardian.com/politics/2021/mar/26/alex‐salmond‐launches‐new‐independence‐focused‐political‐party‐alba

[epdy12362-bib-0005] Green Party (2021). Co‐Leaders: Carla Denyer & Adrian Ramsay. Green Party. Available online at: https://www.greenparty.org.uk/people/leaders‐of‐green‐party.html

[epdy12362-bib-0006] Hancock, M. (2021). Letter from Matt Hancock to the Prime Minister. Available online at: https://www.gov.uk/government/publications/matt‐hancocks‐resignation‐letter‐and‐the‐prime‐ministers‐response

[epdy12362-bib-0007] Peachey, K. (2021). Convicted Post Office workers have names cleared. BBC News online 23 April. Available online at: https://www.bbc.co.uk/news/business‐56859357

[epdy12362-bib-0008] Scott, E. (2022). Vetting appointments to the House of Lords. Available online at: https://lordslibrary.parliament.uk/vetting‐appointments‐to‐the‐house‐of‐lords/

[epdy12362-bib-0009] Southend‐on‐Sea Borough Council (2021). City Status Announced for Southend‐on‐Sea. Southend‐on‐Sea Borough Council. Available online at: https://www.southend.gov.uk/news/article/2382/city‐status‐announced‐for‐southend‐on‐sea

[epdy12362-bib-0010] Survation (2021). New MRP Analysis of Nolan Principles & Westminster Seat Forecast. Survation; Available online at: https://mailchi.mp/survation/new‐survation‐research‐projects‐111‐conservative‐seat‐losses

[epdy12362-bib-0011] UK Parliament (n.d.) . Her Majesty's Government: The Cabinet. UK Parliament; Available online at: https://members.parliament.uk/Government/Cabinet

[epdy12362-bib-0012] UK Parliament (2021a). State of the Parties. UK Parliament; Available online at: https://members.parliament.uk/parties/Commons

[epdy12362-bib-0013] UK Parliament (2021b). Lords Membership. UK Parliament; Available online at: https://members.parliament.uk/parties/Lords

[epdy12362-bib-0014] Vaughan, M. (2021). Dominic Raab Demoted as Foreign Secretary and Replaced by Liz Truss. iNews 15 September. Available online at: https://inews.co.uk/news/politics/dominic‐raab‐demoted‐as‐foreign‐secretary‐and‐replaced‐by‐liz‐truss‐1201091

[epdy12362-bib-0015] YouGov (2021a). Voting Intention 19‐20 Dec. YouGov; Available online at: https://yougov.co.uk/topics/politics/articles‐reports/2021/12/22/voting‐intention‐con‐30‐lab‐36‐19‐20‐dec

[epdy12362-bib-0016] YouGov (2021b). Boris Johnson Approval Rating. YouGov; Available online at: https://yougov.co.uk/topics/politics/trackers/boris‐johnson‐approval‐rating

[epdy12362-bib-0017] YouGov (2021c). Keir Starmer Approval Rating. YouGov; Available online at: https://yougov.co.uk/topics/politics/trackers/keir‐starmer‐approval‐rating?period=all

